# Deciphering the Regulatory Network between the SREBP Pathway and Protein Secretion in *Neurospora crassa*

**DOI:** 10.1128/mBio.00233-17

**Published:** 2017-04-18

**Authors:** Lina Qin, Vincent W. Wu, N. Louise Glass

**Affiliations:** a Plant and Microbial Biology Department, The University of California, Berkeley, California, USA; b The Energy Biosciences Institute, The University of California, Berkeley, California, USA; c Lawrence Berkeley National Laboratory, Berkeley, California, USA; Karlsruhe Institute of Technology (KIT)

## Abstract

Sterol regulatory element binding proteins (SREBPs) are conserved from yeast to mammalian cells and function in the regulation of sterol homeostasis. In fungi, the SREBP pathway has been implicated in the adaptation to hypoxia and in virulence. In *Neurospora crassa* and *Trichoderma reesei*, the SREBP pathway also negatively regulates protein secretion under lignocellulolytic conditions. Here we utilized global transcriptional profiling combined with genetic and physiological analyses to address the regulatory link between the SREBP pathway and protein secretion in *N. crassa*. Our results demonstrated that the function of the SREBP pathway in ergosterol biosynthesis and adaptation to hypoxia was conserved in *N. crassa*. Under lignocellulolytic conditions, the SREBP pathway was highly activated, resulting in the reduced expression of lytic polysaccharide monooxygenases, which require molecular oxygen for catalytic activity. Additionally, activation of the SREBP pathway under lignocellulolytic conditions repressed a set of genes predicted to be involved in the endoplasmic reticulum stress response. Here we show that the inability of a *hac-1* mutant, which bears a deletion of the major regulator of the unfolded protein response (UPR), to efficiently produce cellulases and utilize cellulose was suppressed by mutations in the SREBP pathway. The analyses presented here demonstrated new SREBP pathway functions, including linkages to the UPR, and provide new clues for genetic engineering of filamentous fungi to improve their production of extracellular proteins.

## IMPORTANCE

The role of SREBP transcription factors in the regulation of sterol biosynthesis is conserved from humans to yeast. In filamentous fungi, this pathway regulates the secretion of lignocellulolytic enzymes during plant biomass deconstruction. Here we show that the SREBP pathway in *Neurospora crassa* regulates the production of specific cellulases, lytic polysaccharide monooxygenases that utilize molecular oxygen. Via global transcriptional profile and genetic analyses, a relationship between the SREBP pathway and the unfolded protein response (UPR) pathway was revealed, suggesting a regulatory interplay of these two pathways in the trafficking of plant biomass-degrading enzymes. These findings have implications for our understanding of the cross talk of the SREBP and UPR pathways in other organisms and will guide the rational engineering of fungal strains to improve cellulolytic enzyme production.

## INTRODUCTION

Nonfood sources of plant biomass have been considered promising material for producing renewable biofuels and other bioproducts (1). However, plant biomass-derived products are not currently economically viable partially because of the cost of deconstructing polymers in the plant cell wall to fermentable sugars by lignocellulosic enzymes (2), which are primarily produced by filamentous fungi. The model fungal species *Neurospora crassa* has the ability to degrade plant cell wall material into simple sugars for growth and reproduction. With this property and its well-developed genetic, molecular, and biochemical tools, *N. crassa* has been used to investigate plant cell wall deconstruction by filamentous fungi (3).

To identify unknown factors associated with protein trafficking and secretion in *N. crassa*, ~600 strains carrying deletions in genes predicted to function in the secretory pathway were screened for alterations in the secretion of cellulolytic enzymes (4). The *dsc-2* and *tul-1* (*dsc-1*) mutant hypersecretion strains were identified. The homologs of *dsc-2* and *tul-1* in *Schizosaccharomyces pombe* and *Aspergillus fumigatus* are components of the Golgi apparatus E3 ligase complex critical for the activation of a homolog of the sterol regulatory element binding protein (SREBP) transcription factor, Sre1 or SreA, respectively, by proteolytic cleavage (5, 6). In mammalian cells, the SREBP pathway functions in the regulation of sterol homeostasis. Proteolytic cleavage releases an N-terminally active SREBP from the endoplasmic reticulum (ER) that is subsequently transported into the nucleus to activate the expression of genes associated with cholesterol biosynthesis and lipid metabolism (7). In *N. crassa*, a strain carrying a deletion of the SREBP homolog *sah-2* or mutations in another predicted component of the Golgi apparatus E3 ligase, *dsc-3* or *dsc-4*, show hyperproduction of cellulases (4). A cellulase hyperproduction phenotype was also observed in a *Trichoderma reesei* strain bearing a deletion of a homolog of either *sah-2* or *dsc-1* (4), suggesting a conserved function of the SREBP pathway in the regulation of protein secretion under lignocellulolytic conditions. In *S. pombe*, *A. fumigatus*, and *Cryptococcus neoformans*, in addition to regulation of intracellular sterol homeostasis and adaptation to hypoxic conditions (8–10), the SREBP pathway is associated with the regulation of cell polarity, drug resistance, and virulence (10, 11).

In this study, we further investigated the role of the SREBP pathway in the secretion of lignocellulolytic enzymes by *N. crassa*. We demonstrated that strains carrying mutations in the homologs of SREBP cleavage-activating protein-encoding gene *scp-1* and rhomboid protease-encoding gene *rbd-2* also showed a cellulase hyperproduction phenotype. In addition to regulating ergosterol biosynthesis and hypoxia adaptation, the *N. crassa* SREBP pathway negatively regulated the expression of genes encoding cellobiose dehydrogenase (CDH-1) and most of the lytic polysaccharide monooxygenases (LPMOs), as well as many ER stress-responsive genes. Furthermore, deletion of *sah-2* rescued the cellulase-negative defect of the Δ*hac-1* mutant; HAC-1 is the major regulator of the unfolded protein response (UPR). These findings extend our mechanistic understanding of the regulation of cellulase enzyme secretion and provide a link between the SREBP and UPR pathways in filamentous fungi during plant biomass deconstruction. Additionally, these studies provide new strategies for targeted engineering of filamentous fungi to improve current yields of plant cell wall-degrading enzymes.

## RESULTS

### Identification of components of the SREBP pathway in *N. crassa*.

SREBPs are synthesized in an inactive form and anchored in the ER membrane. In the ER membrane, SREBPs bind to the SREBP cleavage-activating protein Scap, which has a sterol-sensing domain. In sterol-replete cells, the SREBP-Scap complex remains in the ER, with Scap binding to the ER-resident protein INSIG. In sterol-depleted cells, Scap-INSIG binding is disrupted and Scap escorts SREBP to the Golgi apparatus (7). In *S. pombe* and *A. fumigatus*, a Golgi apparatus E3 ligase complex is employed for proteolytic activation of SREBP (5, 6, 12), along with a rhomboid protease, Rbd-2/RbdB (13, 14). In *Aspergillus nidulans* and *A. fumigatus*, a signal peptide peptidase, SppA, is also involved in the cleavage of SREBP (15).

A search of the *N. crassa* genome for genes involved in the SREBP pathway in *Homo sapiens*, *S. pombe*, and *A. fumigatus* showed that *N. crassa* has two homologs of SREBP (*sah-2* and NCU01871). We named NCU01871 *sre-2*. Both *sah-2* and *sre-2* contain a basic helix-loop-helix (bHLH) DNA binding domain in their N terminus and have a tyrosine residue in the bHLH domain, which is a unique characteristic of SREBPs. SAH-2 also has a DUF2014 domain in its C terminus, which is also found in *S. pombe* Sre1 and *A. fumigatus* SrbA, while SRE-2 lacks this C-terminal domain. *N. crassa* has no homolog of the INSIG protein but does have a homolog of Scap1, SppA, and rhomboid protease Rbd-2/RbdB. The homolog of Scap was named *scp-1* (NCU03675); the homologs of the components in the Golgi apparatus E3 ligase complex were named *tul-1* (here *dsc-1*; NCU03740), *dsc-2* (NCU03459), *dsc-3* (NCU11245), *dsc-4* (NCU02676), *dsc-5* (NCU01928), and *dsc-6* (NCU00018). The homolog of the peptide peptidase SppA was named *spp-1* (NCU00568) and the homolog of the rhomboid protease Rbd-2/RbdB was named *rbd-2* (NCU02371). Among these homologs, Δ*scp-1*, Δ*sre-2*, Δ*dsc-3*, and Δ*dsc-6* were not available in the *N. crassa* deletion strain collection. We therefore generated Δ*scp-1* and Δ*sre-2* deletion mutants by replacement of the resident locus with the hygromycin resistance gene *hph* (16) (see Materials and Methods).

**FIG 1:**
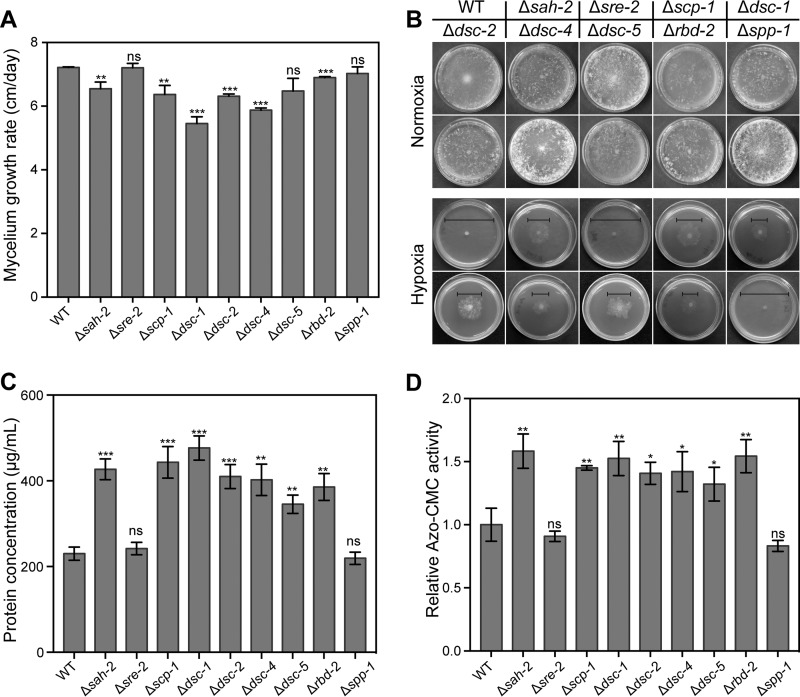
Phenotypic analysis of the potential SREBP-related mutants. (A) Growth rates of the WT and SREBP-related mutants. A suspension of 10^3^ conidia from the strains indicated was inoculated into race tubes containing VMM and incubated for 3 days. Mycelium length was measured every 24 h (B) Growth of the WT and SREBP-related mutants on VMM under normoxic and hypoxic conditions (0.2% O_2_ in N_2_). A suspension of 10^3^ conidia was inoculated into each plate, and growth was observed after 48 h of incubation. The solid line indicates the diameter of each colony. The plates shown represent the replicates of each strain, and the statistical analysis of colony diameter is shown in Fig. S1A. (C, D) Total secreted protein levels (C) and endoglucanase activities (D) in the supernatant of 96-h cultures in VMM with 2% Avicel after a shift from a 16-h VMM culture. Shown are the mean values of three replicates. Error bars show the standard deviations between these replicates. The significance of differences between the mutants and the WT was based on *t* test analysis by the FDR approach. Asterisks indicate significant differences (*, *P* < 0.05; **, *P* < 0.01; ***, *P* < 0.001). ns, not significant. Azo-CMC, azo-carboxymethyl cellulose.

Previously, we determined that deletion mutations in *sah-2*, *dsc-1*, *dsc-2*, and *dsc-4* resulted in strains that showed hyperproduction of cellulases when exposed to cellulose (4). However, the effect of these mutations on the response to hypoxic conditions has not been determined. To determine if the SREBP pathway regulates adaptation to hypoxia in *N. crassa* and if the identified homologs are required, we assessed the growth phenotype of the Δ*sah-2*, Δ*sre-2*, Δ*scp-1*, Δ*dsc-1*, Δ*dsc*-*2*, Δ*dsc-4*, Δ*dsc-5*, Δ*rbd-2*, and Δ*spp-1* mutants under hypoxia and normoxia conditions. The deletion mutants showed somewhat variable growth rates under normoxia conditions, the Δ*dsc-1* and Δ*dsc-4* mutants showed a significantly lower growth rate, while the Δ*sah-2*, Δ*scp-1*, and Δ*rbd-2* mutants grew slightly more slowly (Fig. 1A). The growth rates of the Δ*dsc-5*, Δ*sre-2*, and Δ*spp-1* mutants displayed growth rates that were not statistically significantly different from that of the wild type (WT). However, under hypoxic condition (0.2% O_2_ in N_2_), all but the Δ*sre-2* and Δ*spp-1* mutant strains showed a severe growth defect (Fig. 1B; see Fig. S1A in the supplemental material). Additionally, the hypoxia-sensitive strains showed reduced aerial hyphal growth under normoxia conditions, while the Δ*sre-2* and Δ*spp-1* mutants did not (see Fig. S1B). These data indicate that the SREBP components *scp-1*, the Golgi apparatus E3 ligase complex, *rbd-2*, and *sah-2* have a conserved role in hypoxia adaptation in *N. crassa*, while *sre-2* and *spp-1* do not.

10.1128/mBio.00233-17.1 FIG S1  Phenotypic analysis of SREBP mutants. (A) Colony diameters of SREBP mutants and the WT grown on VMM under hypoxia conditions. (B) SREBP mutants displayed a short aerial hypha growth phenotype. A suspension of 10^5^ conidia was inoculated into each flask, and growth was observed after 4 days of incubation. (C) Growth of M^*clr-*2^ Δ*sah-2*, M^*clr-*2^ Δ*dsc-1*, M^*clr-*2^ Δ*scp-1*, and M*clr-2* on VMM under normoxic and hypoxic conditions. A suspension of 10^3^ conidia was inoculated onto each plate, and growth was observed after 48 h of incubation. The solid line indicates the diameter of each colony. (D) Colony diameters of M^*clr-*2^ Δ*sah-2*, M^*clr-*2^ Δ*dsc-1*, M^*clr-*2^ Δ*scp-1*, and M*clr-2* strains grown on VMM under hypoxic conditions. (E) Growth rates of M^*clr-*2^ Δ*sah-2*, M^*clr-*2^ Δ*dsc-1*, M^*clr-*2^ Δ*scp-1*, and M*clr-2* under normoxic conditions. A suspension of 10^3^ conidia from each strain was inoculated into race tubes containing VMM and incubated for 3 days. Mycelium length was measured every 24 h. (F) Morphological phenotypes of SREBP mutants versus that of the M*clr-2* strain on VMM slants. Asterisks indicate significant differences (*, *P* < 0.05; **, *P* < 0.01; ***, *P* < 0.001). ns, not significant. Download FIG S1, TIF file, 2.6 MB. Copyright © 2017 Qin et al. 2017 Qin et al. This content is distributed under the terms of the Creative Commons Attribution 4.0 International license.

To further confirm the hypothesis that any strain containing a loss-of-function mutation in a component of the SREBP pathway would show a cellulolytic hyperproduction phenotype, we compared the total secreted protein level and endoglucanase activity under Avicel conditions of the nine predicted SREBP pathway deletion strains with those of the WT strain. As predicted, these results showed that the total secreted protein level and endoglucanase activity of the Δ*sah-2*, Δ*scp-1*, Δ*dsc-1*, Δ*dsc-2*, Δ*dsc-4*, Δ*dsc-5*, and Δ*rbd-2* mutants were significantly higher than those of the WT strain (Fig. 1C and D). In contrast, the two mutants that showed no defect in growth under hypoxic conditions, the Δ*sre-2* and Δ*spp-1* mutants, also showed no hyperproduction of cellulases. Taken together, these data indicated that the SREBP pathway, which is important for the response of *N. crassa* to hypoxic conditions, also plays a role in the regulation of the production/secretion of cellulolytic enzymes.

### Ergosterol levels are reduced in SREBP-related mutants.

**FIG 2:**
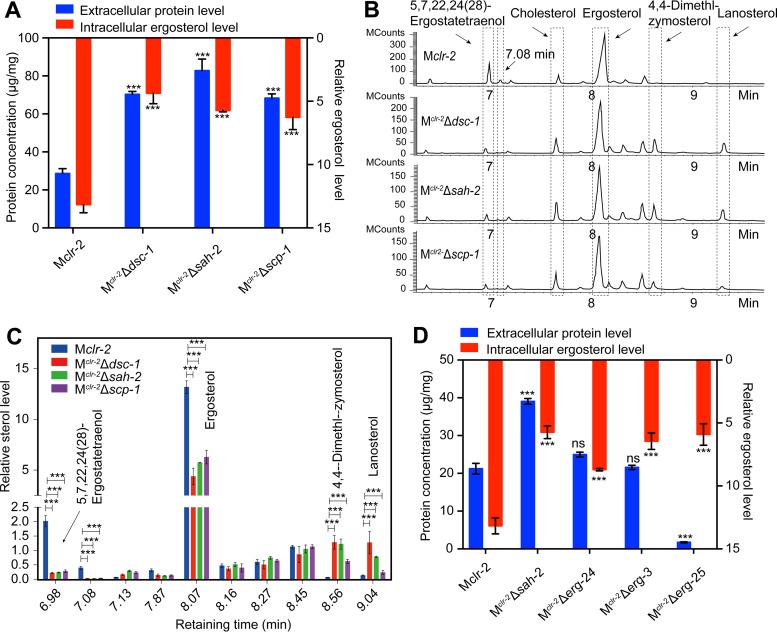
SREBP-regulated ergosterol biosynthesis pathway in *N. crassa*. (A) Total secreted protein level and intracellular ergosterol levels in a 48-h VMM culture of the strains indicated after a shift from a 16-h VMM culture. (B) GC-MS chromatogram of sterol extracts from the strains indicated. MCounts, mega counts, indicating the intensity (abundance) of the signal for each peak. (C) Quantified level of each sterol component in extracts of M*clr-2*, M^*clr-*2^ Δ*dsc-1*, M^*clr-*2^ Δ*sah-2*, and M^*clr-*2^ Δ*scp-1* cultures. The relative level of each sterol component was normalized by cholesterol internal standard. (D) Total secreted protein level and intracellular ergosterol levels of the strains indicated under the same conditions as for panel A.

To determine whether the SREBP pathway plays a role in sterol homeostasis and to determine if this role is involved in the cellulase hyperproduction phenotype in *N. crassa*, we crossed three key SREBP pathway mutants, the Δ*sah-2*, Δ*dsc-1*, and Δ*scp-1* mutants, with a strain that constitutively expresses the major transcriptional regulator of cellulolytic genes (*Pccg-1-clr-2*; termed M*clr-2*) (17), resulting in strains M^*clr-*2^ Δ*sah-2*, M^*clr-*2^ Δ*dsc-1*, and M^*clr-*2^ Δ*scp-1*. We constructed strains containing this *clr-2* construct (*Pccg-1-clr-2*), as the production of cellulases is inducer independent and the cellulase hyperproduction phenotype of the Δ*dsc-1* and Δ*dsc-2* mutants was conserved in these strains (4). Consistent with the phenotype of the single-deletion mutants, the M^*clr-*2^ Δ*sah-2*, M^*clr-*2^ Δ*dsc-1*, and M^*clr-*2^ Δ*scp-1* strains grew slightly more slowly than the M*clr-2* strain, showed reduced aerial hyphae when grown on sucrose under normoxic conditions, and displayed growth defects under hypoxic conditions (see Fig. S1C to F). To assess the relationship between protein secretion and ergosterol in these mutants, the total secreted protein levels and intracellular ergosterol levels in the M^*clr-*2^ Δ*sah-2*, M^*clr-*2^ Δ*dsc-1*, and M^*clr-*2^ Δ*scp-1* strains were compared to those in the M*clr-2* strain. As expected, the protein secretion levels in the M^*clr-*2^ Δ*sah-2*, M^*clr-*2^ Δ*dsc-1*, and M^*clr-*2^ Δ*scp-1* strains were significantly increased. However, ergosterol levels were significantly lower in the SREBP-related mutants than in the M*clr-2* strain (Fig. 2A). Moreover, gas chromatography (GC)-mass spectrometry (MS) showed that the pattern abundances of the sterol intermediates in the SREBP-related mutants were very similar (Fig. 2B and C) to those of the M*clr-2* strain but with important key differences. For example, two ergosterol intermediates, 4,4-dimethl-zymosterol and lanosterol, accumulated in the SREBP-related mutants, while 5,7,22,24(28)-ergostatetraenol and an unidentified component with a retaining time of 7.08 min were markedly lower than in the M*clr-2* strain. To determine whether SREBP has a role in ergosterol biosynthesis under noncellulosic conditions, we compared the levels of ergosterol and its intermediates in the Δ*sah-2* mutant and WT strains under sucrose conditions (see Fig. S2). The results showed that the sterol pattern in the Δ*sah-2* mutant was similar to that of the M^*clr-*2^ Δ*sah-2*, M^*clr-*2^ Δ*dsc-1*, and M^*clr-*2^ Δ*scp-1* strains (Fig. 2; see Fig. S2). These data indicated that the SREBP pathway regulates egosterol homeostasis in *N. crassa*, as observed in other fungi.

10.1128/mBio.00233-17.2 FIG S2  SREBP-regulated ergosterol biosynthesis pathway in *N. crassa*. (A) The quantified level of each sterol component in extracts of the WT and Δ*sah-2* mutant strains in 48-h VMM cultures after a shift from 16-h VMM cultures. (B) The GC-MS chromatogram of sterol extracts from the WT and Δ*sah-2* mutant strains. (C) Total secreted protein levels in a 16-h VMM M*clr-2* culture shifted to treatment with fluconazole for 48 h at the concentration indicated. Asterisks indicate significant differences (*, *P* < 0.05; **, *P* < 0.01; ***, *P* < 0.001). ns, not significant. Download FIG S2, TIF file, 0.8 MB. Copyright © 2017 Qin et al. 2017 Qin et al. This content is distributed under the terms of the Creative Commons Attribution 4.0 International license.

Ergosterol is a component of fungal cell membranes, and the concentration of sterols within eukaryotic membranes affects many membrane-associated functions, including vesicle formation and protein sorting (18). For example, blocking of ergosterol synthesis with the antifungal agent fluconazole results in increased heterologous recombinant protein production in the yeast *Pichia pastoris* (19). To determine whether the hypersecretion phenotype observed in SREBP-related mutants resulted from lower ergosterol content, we constructed Δ*erg-25* mutant strains by replacing the resident gene with the nourseothricin acetyltransferase gene *nat1* (see Materials and Methods). The Δ*erg-25* deletions, along with the Δ*erg-3* and Δ*erg-24* deletions (available from the *N. crassa* deletion collection), were introduced into the M*clr-2* strain via crosses. Intracellular ergosterol levels and the total secreted protein levels of the M^*clr-*2^ Δ*erg-3*, M^*clr-*2^ Δ*erg-24*, and M^*clr-*2^ Δ*erg-25* strains under sucrose conditions were measured. As expected, the ergosterol levels in all three mutants were significantly lower than those in the M*clr-2* strain. However, the levels of secreted proteins were not increased in any of the ergosterol mutants in the M*clr-2* background (Fig. 2D). As an alternative approach to explore the relationship between ergosterol levels and protein secretion, we used fluconazole, a specific inhibitor of the essential protein ERG-11, to treat the M*clr-2* strain under sucrose conditions. As shown in Fig. S2C, treatment with fluconazole also did not increase secreted protein levels. These data indicated that the hyperproduction of proteins observed in the SREBP-related mutants under cellulolytic conditions did not result from disruption of the ergosterol biosynthesis pathway.

### Transcriptional profiling revealed that the SREBP pathway is highly activated under lignocellulolytic conditions.

To gain insight into the molecular basis of the relationship between the SREBP pathway and hyperproduction of cellulases, we conducted transcriptome sequencing (RNA-seq) to compare global changes in gene expression levels among the WT, Δ*sah-2* mutant, M*clr-2*, and M^*clr-*2^ Δ*sah-2* strains grown in sucrose minimal medium. Under this condition, cellulase gene expression is repressed in the WT and Δ*sah-2* mutant strains but induced in the M*clr-2* and M^*clr-*2^ Δ*sah-2* strains. Principal-component analysis (PCA) and Euclidean distance analyses of the 12 sets of RNA-seq data showed that the biological replicate samples clustered together and that the expression pattern of M^*clr-*2^ Δ*sah-2* was most similar to that of the Δ*sah-2* mutant (see Fig. S3). Numbers of reads per kilobase of transcript per million mapped reads (RPKM) for all genes were calculated for biological replicates, and differential gene expression analyses (≥4-fold change; *P* ≤ 0.01) between Δ*sah-2* mutant and WT, M*clr-2* and WT, M^*clr-*2^ Δ*sah-2* and M*clr-2*, and M^*clr-*2^ Δ*sah-2* and Δ*sah-2* mutant samples were performed (see Fig. S4 and Data Set S1).

10.1128/mBio.00233-17.3 FIG S3  Triplicates of RNA-seq samples cluster within each strain. PCA (A) and Euclidean distance analysis (B) of the 12 sets of RNA-seq data from the WT, M*clr-2*, Δ*sah-2* mutant, and M^*clr-*2^ Δ*sah-2* strains cultured for 24 h in VMM after a shift from 16-h-old VMM cultures. Download FIG S3, TIF file, 1.9 MB. Copyright © 2017 Qin et al. 2017 Qin et al. This content is distributed under the terms of the Creative Commons Attribution 4.0 International license.

10.1128/mBio.00233-17.4 FIG S4  Comparison of gene expression data sets. Comparison of gene expression in the Δ*sah-2* mutant versus the WT (A), in M*clr-2* versus the WT (B), in M^*clr-*2^ Δ*sah-2* versus M*clr-2* (C), and in M^*clr-*2^ Δ*sah-2* versus the Δ*sah-2* mutant (D). Log_2_-fold changes in gene expression versus the mean of normalized read counts plotted by the DeSeq package (43, 44). Red dots indicate significant differential expression of genes with an adjusted *P* value of <0.01. Green dots indicate CLR-2 direct targeted genes as determined by ChIP-seq analyses (44). Download FIG S4, TIF file, 2 MB. Copyright © 2017 Qin et al. 2017 Qin et al. This content is distributed under the terms of the Creative Commons Attribution 4.0 International license.

10.1128/mBio.00233-17.8 DATA SET S1  Sheet 1, differential-expression analysis of the Δ*sah-2* mutant versus the WT. Sheet 2, differential-expression analysis of M*clr-2* versus the WT. Sheet 3, differential-expression analysis of M*clr-2* Δ*sah-2* versus M*clr-2*. Sheet 4, differential-expression analysis of M*clr-2* Δ*sah-2* versus the Δ*sah-2* mutant. Sheet 5, three-way Venn diagram of genes upregulated in the Δ*sah-2* mutant versus the WT, in M*clr-2* Δ*sah-2* versus M*clr-2*, and in M*clr-2* Δ*sah-2* versus the Δ*sah-2* mutant. Sheet 6, three-way Venn diagram of genes downregulated in the Δ*sah-2* mutant versus the WT, in M^*clr-*2^ Δ*sah-2* versus M*clr-2*, and in M^*clr-*2^ Δ*sah-2* versus the Δ*sah-2* mutant. Sheet 7, FunCat analysis of the upregulated 236-gene set. Sheet 8, FunCat analysis of the upregulated 241-gene set. Sheet 9, FunCat analysis of the upregulated 571-gene set. Sheet 10, list of the 176 protein secretion genes regulated in M^*clr−*2^ Δ*sah-2* versus M*clr-2*. Download DATA SET S1, XLSX file, 0.6 MB. Copyright © 2017 Qin et al. 2017 Qin et al. This content is distributed under the terms of the Creative Commons Attribution 4.0 International license.

**FIG 3:**
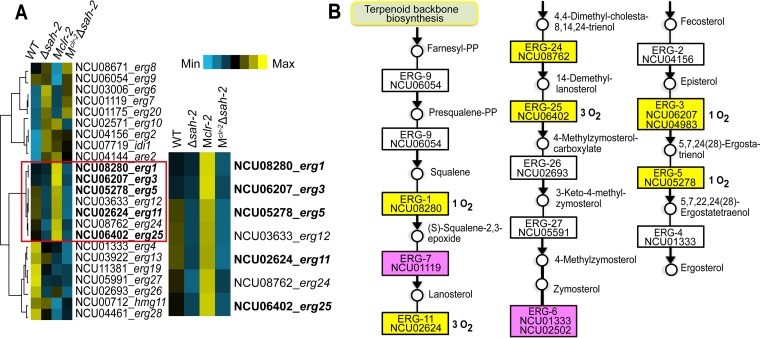
Influence of *sah-2* on the expression of genes involved in the ergosterol biosynthesis pathway. (A) Hierarchical clustering of genes involved in ergosterol biosynthesis. Results are displayed as heat maps with log numbers of RPKM ranging from minimum (bright blue) to maximum (bright yellow). The SREBP direct targets, as identified in *A. fumigatus* (30), are shown in bold at the right. (B) Ergosterol biosynthesis pathway. The genes highlighted in yellow were those whose expression levels were downregulated both in the Δ*sah-2* mutant versus the WT and in the M^*clr-*2^ Δ*sah-2* strain versus the M*clr-2* strain. The genes highlighted in pink were those whose expression levels were upregulated both in the Δ*sah-2* mutant versus the WT and in the M^*clr-*2^ Δ*sah-2* strain versus the M*clr-2* strain. Oxygen-requiring enzymes are marked.

Hierarchical clustering of genes involved in ergosterol biosynthesis revealed that *erg-1* (NCU08280), *erg-11* (NCU02624), *erg-3* (NCU06207), *erg-25* (NCU06402), and *erg-5* (NCU05278) showed significantly lower expression levels in the Δ*sah-2* mutant compared to the WT and in M^*clr-*2^ Δ*sah-2* compared to M*clr-2* (Fig. 3A). Most of these ergosterol biosynthetic genes encode oxygen-dependent enzymes (Fig. 3B). We further explored the *erg-3*, *erg-11*, *erg-24*, and *erg-25* gene expression levels in the M^*clr-*2^ Δ*dsc-1*, M^*clr-*2^ Δ*scp-1*, M^*clr-*2^ Δ*sah-2*, and M*clr-2* strains by quantitative reverse transcription (qRT)-PCR. As with the RNA-seq analyses of M^*clr-*2^ Δ*sah-2*, these data showed that the ergosterol biosynthetic gene expression levels were also significantly decreased in the Δ*dsc-1* and Δ*scp-1* mutant strains, indicating that the SREBP pathway is required for proper expression of ergosterol biosynthetic genes (see Fig. S5A).

10.1128/mBio.00233-17.5 FIG S5  The SREBP pathway is activated under lignocellulase gene expression conditions. (A) qRT-PCR analysis of *erg-3*, *erg-11*, *erg-24*, and *erg-25* gene expression of M^*clr-*2^ Δ*sah-2*, M^*clr-*2^ Δ*dsc-1*, and M^*clr-*2^ Δ*scp-1* cultured for 24 h in VMM following a shift from 16-h-old VMM cultures. (B) qRT-PCR analysis of *erg-3*, *erg-11*, and *erg-25* expression levels in the WT, M*clr-2*, and M^*clr-*2^ Δ*sah-2* strains from VMM cultures for the times indicated after a shift from 16-h-old VMM cultures. (C) Total extracellular protein levels in the supernatants of VMM cultures of M*clr-2* and M^*clr-*2^ Δ*sah-2* grown in a 1-liter bioreactor with or without DO control. Asterisks indicate significant differences (**, *P* < 0.01; ***, *P* < 0.001). ns, not significant. Download FIG S5, TIF file, 1.6 MB. Copyright © 2017 Qin et al. 2017 Qin et al. This content is distributed under the terms of the Creative Commons Attribution 4.0 International license.

In the M*clr-2* strain, the *erg-1*, *erg-11*, *erg-3*, *erg-25*, and *erg-5* expression levels were strongly increased relative to those in the WT strain, suggesting that the induction of cellulases affected SAH-2 activation and thus expression of the *erg* genes (Fig. 3A). Importantly, the *clr-2* transcript abundance in M^*clr-*2^ Δ*sah-2* was not higher than in M*clr-2*, indicating that the hyperproduction of cellulases in M^*clr-*2^ Δ*sah-2* was not due to an increase in *clr-2* expression. To test the hypothesis that the induction of cellulases is associated with the activation of SAH-2, we performed qRT-PCR to examine the *erg-3*, *erg-11*, and *erg-25* mRNA abundance in the WT, M*clr-2*, Δ*sah-2* mutant, and M^*clr-*2^ Δ*sah-2* strains at different time points. These data showed that the *erg* gene expression levels in the M*clr-2* strain were higher at 24 and 48 h (see Fig. S5B) than at the 4-h time point. Since these genes are predicted to be directly regulated by SAH-2 (see Discussion), this observation indicated that the SREBP pathway was activated during the induction and trafficking of cellulases, which are associated with deconstruction of plant biomass.

In *S. pombe* and *A. fumigatus*, the activation of orthologs of SREBP (Sre1 and SreA, respectively) is triggered by hypoxia. This observation suggested that the trafficking and secretion of cellulases might result in reduction of oxygen availability in cells, resulting in activation of the SREBP pathway. To test this hypothesis, we performed growth experiments with parallel bioreactor fermenters containing a dissolved oxygen (DO) control system (see Materials and Methods). Consistent with this hypothesis, the increased protein secretion in M^*clr-*2^ Δ*sah-2* was only observed when the automatic DO control system in the fermenter was turned off. The levels of protein secretion in the M^*clr-*2^ Δ*sah-2* and M*clr-2* strains were not significantly different when the automatic DO control system was turned on (see Fig. S5C). These data suggest that cellulase production leads to higher oxygen consumption, thereby lowering the oxygen level in the surrounding environment and resulting in activation of the SREBP pathway.

### CDH-1 and LPMO gene expression levels increase in the SREBP mutant.

**FIG 4:**
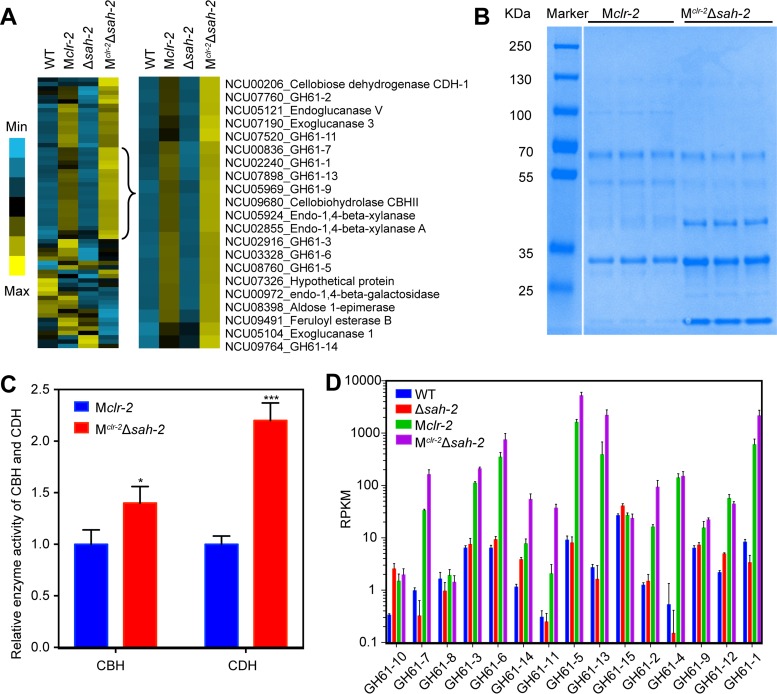
Strains bearing a deletion of *sah-2* show increased expression of genes encoding LPMOs. (A) Hierarchical clustering of genes encoding extracellular proteins. Results are displayed as heat maps with log numbers of RPKM ranging from minimum (bright blue) to maximum (bright yellow). Genes with a higher expression level in the M^*clr-*2^ Δ*sah-2* strain than in the M*clr-2* strain are highlighted at the right. (B) SDS-PAGE analysis of the supernatants of the M*clr-2* and M^*clr-*2^ Δ*sah-2* strains from three independent 16-h cultures shifted for 48 h to VMM. (C) Measurement of the total CBH-1 and CDH-1 enzyme activities in the supernatant of M*clr-2* and M^*clr-*2^ Δ*sah-2* after 48 h of growth in VMM. (D) The expression levels (RPKM) of 15 currently identified *N. crassa* LPMO-encoding genes in the WT, M*clr-2*, the Δ*sah-2* mutant, and M^*clr-*2^ Δ*sah-2*. Asterisks indicate significant differences (*, *P* < 0.05; ***, *P* < 0.001).

Our previous data showed that deletion of *sah-2* or *dsc-1* does not significantly affect the expression level of the major cellulase genes *cbh-1*, *cbh-2*, and *gh5-1* (4). To determine if the expression of genes encoding other extracellular proteins was affected in the SREBP pathway mutants, we performed hierarchical clustering of genes encoding proteins identified as extracellular when *N. crassa* was exposed to Avicel or *Miscanthus* (20). This analysis showed that the expression of ~2/3 of this gene set is CLR-2 dependent and 20 of them showed higher expression levels in the M^*clr-*2^ Δ*sah-2* strain than in the M*clr-2* strain (Fig. 4A; see Table S1). Ten of these 20 genes encode LPMO enzymes. LPMOs are copper-dependent redox enzymes that require molecular oxygen and an external electron donor to cleave glycosidic bonds (21). CDH-1, the key electron donor for LPMOs (22), showed 12-fold higher expression in the M^*clr-*2^ Δ*sah-2* strain than in the M*clr-2* strain. Consistent with the RNA-seq data, SDS-PAGE analysis of the supernatants of the M^*clr-*2^ Δ*sah-2* and M*clr-2* strains showed variations in specific protein levels rather than an overall increase in the levels of secreted proteins in the M^*clr-*2^ Δ*sah-2* strain (Fig. 4B). We thus measured the extracellular activity of cellobiohydrolase (CBH-1; *cbh-1* expression was not significantly affected in the Δ*sah-2* mutant) and CDH-1 (*cdh-1* expression increased in the Δ*sah-2* mutant) from the M^*clr-*2^ Δ*sah-2* and M*clr-2* strains. These data showed that the activity of CDH-1 was 2.2-fold higher and that of CBH-1 was 1.4-fold higher in the M^*clr-*2^ Δ*sah-2* strain than that in the M*clr-2* strain (Fig. 4C). These data suggested that the expression level of genes and thus the activity of the enzymes associated with oxygen consumption under lignocellulolytic conditions are negatively regulated by SAH-2.

10.1128/mBio.00233-17.6 TABLE S1  Expression levels of extracellular protein-encoding genes with higher expression levels in M^*clr-*2^ Δ*sah-2* than in *Mclr-2*. Download TABLE S1, DOCX file, 0.02 MB. Copyright © 2017 Qin et al. 2017 Qin et al. This content is distributed under the terms of the Creative Commons Attribution 4.0 International license.

There are 15 LPMO genes predicted in the *N. crassa* genome. To determine if SAH-2 influences the expression of the remaining five LPMOs, we assessed the expression levels of these remaining LPMO genes in the WT, M*clr-2*, Δ*sah-2* mutant, and M^*clr-*2^ Δ*sah-2* strains. While the expression of two predicted LPMO genes, *gh61-8* (NCU03000) and *gh61-15* (NCU07974), was not affected by the deletion of *sah-2*, the expression levels of two additional LPMO genes, *gh61-10* (NCU01867) and *gh61-12* (NCU02344), were 7.6- and 2.3-fold higher, respectively, in the Δ*sah-2* mutant than in the WT (Fig. 4D). Thus, deletion of *sah-2* resulted in a significant increase in the expression levels of 12 out of 15 LMPO genes identified in the genome of *N. crassa*. These data indicate that under low-oxygen conditions, SAH-2 is required for activation of the expression of genes required for sterol synthesis and adaptation to hypoxia, but the expression of genes encoding extracellular proteins, such as LPMOs, whose function requires molecular oxygen, is repressed.

### Genes involved in ER stress responses showed increased expression levels in the SREBP mutant.

**FIG 5:**
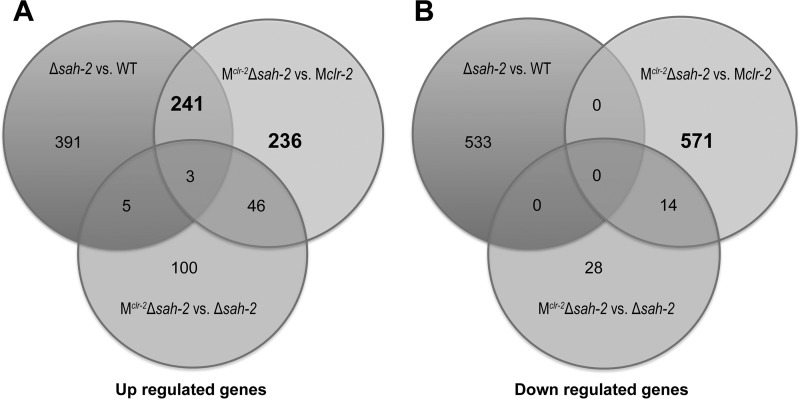
Analysis of *sah-2* regulons. (A) Three-way Venn diagram pooling genes upregulated in the Δ*sah-2* mutant versus the WT, in M^*clr-*2^ Δ*sah-2* versus M*clr-2*, and in M^*clr-*2^ Δ*sah-2* versus the Δ*sah-2* mutant. The threshold of differential gene expression was set as a ≥4-fold change (*P* ≤ 0.01). (B) Three-way Venn diagram pooling genes downregulated in the Δ*sah-2* mutant versus the WT, in M^*clr-*2^ Δ*sah-2* versus M*clr-2*, and in M^*clr-*2^ Δ*sah-2* versus the Δ*sah-2* mutant. The threshold of differential gene expression was set as a ≥4-fold change (*P* ≤ 0.01).

Considering the substantial cellulase hyperproduction phenotype observed in all of the SREBP pathway mutants, we asked whether SAH-2 also has a role in the modulation of protein secretion. We thus evaluated genes with expression changes (≥4-fold changes, *P* ≤ 0.01) in the Δ*sah-2* mutant RNA-seq data compared to those of the WT, as well as in the M^*clr-*2^ Δ*sah-2* RNA-seq data set compared to those of the M*clr-2* strain. These analyses showed that 396 genes were upregulated and 533 genes were downregulated in the Δ*sah-2* mutant compared to those in the WT, while 282 genes were upregulated and 585 genes were downregulated in the M^*clr-*2^ Δ*sah-2* strain compared to those in M*clr-2*. In these gene sets, 244 genes in the upregulated sets overlapped, while no overlap was detected in the downregulated gene sets (Fig. 5A and B; see Data Set S1).

To search for genes that are predicted to affect protein secretion but are not regulated directly by CLR-2, we focused on the 477 genes (236 plus 241; Fig. 5A) whose expression level increased in the M^*clr-*2^ Δ*sah-2* strain relative to that in the M*clr-2* strain and the 571 genes whose expression level decreased in the M^*clr-*2^ Δ*sah-2* strain relative to that in the M*clr-2* strain (Fig. 5B). FunCat (23) analysis of the set of 236 upregulated genes revealed that 80 genes for cellular transport, transport facilities, and transport routes were enriched (*P* = 4.87E-05); 60 genes involved in protein folding, modification, and destination were enriched (*P* = 0.01); and 20 genes involved in cellular export and secretion were enriched (*P* = 0.02) (see Data Set S1). FunCat analysis of the set of 241 upregulated genes showed that 71genes involved in cellular transport, transport facilities, and transport routes were enriched (*P* = 0.002) and 14 genes involved in protein folding and stabilization were enriched (*P* = 0.01). A comparison of these two gene sets revealed that a total of 176 protein secretion-associated genes showed higher expression levels in the M^*clr-*2^ Δ*sah-2* strain than in the M*clr-2* strain (see Data Set S1). In contrast, no protein secretion-associated functional categories were enriched in the set of 571 downregulated genes.

**FIG 6:**
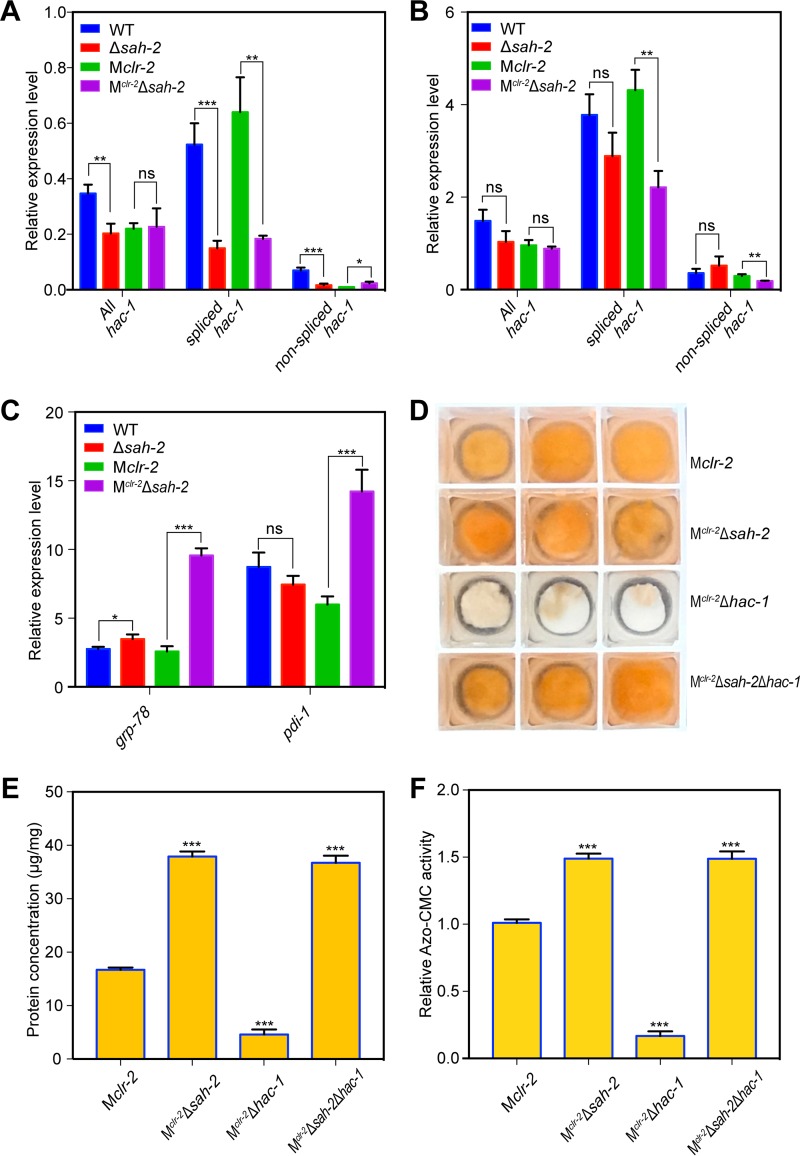
The upregulated UPR in M^*clr-*2^ Δ*sah-2* is independent of *hac-1* regulation. (A) Relative quantification of the amount of *hac-1* mRNA splice variants in the WT, M*clr-2*, Δ*sah-2* mutant, and M^*clr-*2^ Δ*sah-2* strains by qRT-PCR from samples grown for 24 h in VMM following a shift (16-h culture grown in VMM). (B) Fold changes in the expression levels of *hac-1* mRNA splice variants with DTT treatment. RNA samples were obtained from cultures grown in VMM or in VMM plus 10 mM DTT for 4 h after a shift of 16-h-old VMM cultures. (C) Fold change in the expression levels of *grp-78* and *pdi-1* in cultures treated with DTT. RNA was isolated from cultures as described for panel B. The results in panels B and C are fold induction in a 4-h culture in VMM plus 10 mM DTT over the expression state in a 4-h VMM culture. (D) Growth phenotypes of the M*clr-2*, M^*clr-*2^ Δ*sah-2*, M^*clr-*2^ Δ*hac-1*, and M^*clr-*2^ Δ*sah-2* Δ*hac-1* strains in VMM with 2% Avicel for 96 h. (E, F) Total secreted protein level (E) and endoglucanase activities (F) of M*clr-2*, M^*clr-*2^ Δ*sah-2*, M^*clr-*2^ Δ*hac-1*, and M^*clr-*2^ Δ*sah-2* Δ*hac-1* strains in the supernatant of 48-h VMM cultures after a shift from 16-h VMM cultures. Protein concentration and endoglucanase activity were normalized by total fungal cell biomass in each culture. Asterisks indicate significant differences (*, *P* < 0.05; **, *P* < 0.01; ***, *P* < 0.001). ns, not significant.

Investigation of the 176-gene set enriched in protein secretion functional categories revealed that the expression levels of many genes encoding heat shock proteins, such as *hsp30* (NCU09364), *hsp70-5* (NCU08693), *hsp98* (NCU00104), and *hsp60* (NCU01589), were significantly higher in the M^*clr-*2^ Δ*sah-2* strain than in the M*clr-2* strain. These proteins are typically involved in the UPR; activation of the UPR is an indicator of ER stress (24). We therefore evaluated the expression level of genes encoding proteins potentially involved in the UPR, including chaperones, foldases, glycosylation enzymes, vesicle transport proteins, and ER-associated degradation proteins (25, 26), in the M^*clr-*2^ Δ*sah-2* strain compared to those in the M*clr-2* strain. As shown in Table S2, the expression levels of many ER stress response genes, such as *grp-78* (NCU03928) and *pdi-1* (NCU09223), were >2-fold higher in the M^*clr-*2^ Δ*sah-2* strain than in the M*clr-2* strain. However, the expression levels of *hac*-*1* and *ire-1*, whose major function is activation of the UPR pathway (26), were not increased. A key attribute used to assess activation of the UPR is the nonspliceosomal splicing reaction of the *hac-1* mRNA, which results in the removal of a 23-nucleotide unconventional intron and subsequent translational activation of HAC-1 (27). We therefore used qRT-PCR to assess the expression levels of total *hac-1* transcripts, spliced *hac-1* transcripts, and nonspliced *hac-1* transcripts with primer sets described previously (27). As shown in Fig. 6A, the expression levels of total *hac-1* transcripts were significantly lower in the M*clr-2*, Δ*sah-2* mutant, and M^*clr-*2^ Δ*sah-2* strains than in the WT strain. The spliced *hac-1* transcript levels were also significantly lower in both the Δ*sah-2* mutant and M^*clr-*2^ Δ*sah-2* strains, suggesting that the activation of genes involved in ER stress and the UPR in the M^*clr-*2^ Δ*sah-2* strain may occur by an alternative pathway.

10.1128/mBio.00233-17.7 TABLE S2  Expression levels of genes involved in ER stress responses in the WT, M*clr-2*, Δ*sah-2* mutant, and M^*clr-*2^ Δ*sah-2* strains. Download TABLE S2, DOCX file, 0.03 MB. Copyright © 2017 Qin et al. 2017 Qin et al. This content is distributed under the terms of the Creative Commons Attribution 4.0 International license.

The reducing agent dithiothreitol (DTT) induces ER stress by altering the ER oxidative status and disulfide bond formation, leading to the accumulation of misfolded proteins; exposure to DTT triggers UPR in *N. crassa* (26, 27). To further investigate the role of *sah-2* in the UPR pathway, 16-h-old cultures of the WT, M*clr-2*, Δ*sah-2* mutant, and M^*clr-*2^ Δ*sah-2* strains were transferred to either fresh Vogel’s minimal medium (VMM) or VMM plus 10 mM DTT for 4 h. RNA was collected from these cultures, and qRT-PCR was performed to evaluate the splicing of *hac-1* mRNA and the expression levels of the UPR marker genes *grp-78* (NCU03928) and *pdi-1* (NCU09223). In contrast to those of strains grown in VMM, the level of *hac-1* transcripts was significantly induced when cultures were exposed to DTT, but the level of spliced *hac-1* was much lower in the M^*clr-*2^ Δ*sah-2* strain than in the M*clr-2* strain (Fig. 6B). Both UPR marker genes *grp-78* and *pdi-1* were significantly induced in all four strains after treatment with DTT, but the level of induction in M^*clr-*2^ Δ*sah-2* was significantly higher than that in the other three strains (Fig. 6C).

It has been reported that a deletion of *hac-1* in *N. crassa* results in a strain that shows a greatly reduced ability to utilize cellulose and secrete lignocellulolytic enzymes (26, 28). This observation suggested that the inability to mount an appropriate UPR results in an inability to utilize cellulose because of a dysfunction in the proper trafficking of lignocellulolytic enzymes. Because the M^*clr-*2^ Δ*sah-2* and Δ*sah-2* mutant strains showed an increase in the expression levels of genes involved in protein trafficking and the UPR (see Table S2) yet showed a reduction in spliced *hac-1*, we hypothesized that deletion of *sah-2* in a Δ*hac-1* mutant would restore the utilization of cellulose and cellulase secretion. We first tested whether a strain carrying the Δ*hac-1* mutation in the M*clr-2* background (M^*clr-*2^ Δ*hac-1*) failed to secrete cellulases under minimal medium conditions. Indeed, the M^*clr-*2^ Δ*hac-1* mutant showed much lower protein secretion and cellulase activity in VMM than the M*clr-2* strain (Fig. 6E and F). These data indicated that the Δ*hac-1* mutant’s cellulase-deficient phenotype was independent of any inducer (Avicel). We further constructed a Δ*hac-1* Δ*sah-2* mutant in the M*clr-2* background (M^*clr-*2^ Δ*sah-2* Δ*hac-1*). In contrast to the M^*clr-*2^ Δ*hac-1* strain, and in support of our hypothesis, the M^*clr-*2^ Δ*sah-2* Δ*hac-1* strain showed robust protein secretion and cellulase activity that was indistinguishable from that of the M^*clr-*2^ Δ*sah-2* strain (Fig. 6E and F). The phenotype of the M^*clr-*2^ Δ*sah-2* Δ*hac-1* strain was also indistinguishable from that of the M*clr-2* and M^*clr-*2^ Δ*sah-2* strains under Avicel conditions but markedly different from the cellulase-deficient phenotype of the M^*clr-*2^ Δ*hac-1* strain (Fig. 6D).

## DISCUSSION

Extensive studies of the SREBP pathway in a variety of organisms have revealed that, in addition to regulating lipid homeostasis, the SREBP pathway plays additional roles in the regulation of cell polarity, antifungal drug resistance, and virulence (10, 11, 29). Although *N. crassa* has homologs of the SREBP components identified in *S. pombe* and *A. fumigatus*, its role in the biology of this organism has not been extensively studied. Phenotypic analysis of strains containing mutations in SREBP components showed that this pathway is important for both mediation of the cellular response to hypoxia and regulation of ergosterol biosynthesis (Fig. 1; see Fig. S1). As shown for *A. fumigatus*, it is likely that *scp-1*, the Golgi apparatus E3 ligase Dsc complex, and the rhomboid protease-encoding gene *rbd-2* are required for the activation of SAH-2 in *N. crassa*. However, neither the second homolog of SREBP, *sre-2*, nor *spp-1* was required for SREBP pathway function in *N. crassa*. In *A. nidulans*, SppA, which is an aspartyl protease involved in regulated intramembrane proteolysis, is essential for adaptation to hypoxia (15), while in *A. fumigatus*, SrbB, which has homology to SrbA (SREBP), is also required for the response to hypoxia and for virulence (30). Our data also support a role for SREBP in the secretion of lignocellulolytic enzymes in *N. crassa*.

**FIG 7:**
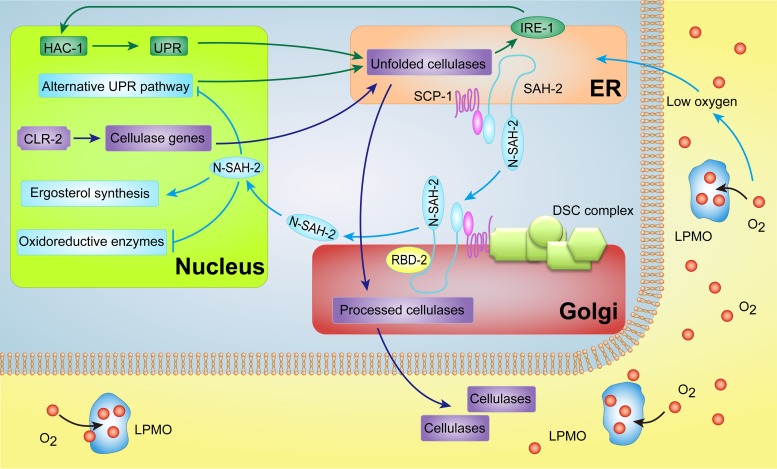
Proposed model of SREBP modulation of cellulase secretion in *N. crassa*. Genes encoding cellulases, including the LPMO genes, are induced by CLR-2 in response to the presence of plant biomass. The IRE-1/HAC-1-mediated UPR is triggered to meet the demand for protein folding in the ER during the trafficking of cellulases through the secretion pathway. The SREBP pathway is activated when the cells sense a low-oxygen environment, perhaps partially due to the demands of cellulase trafficking and contributed by oxygen-consuming extracellular enzymes, such as LPMOs. The ER-bond SAH-2-SCP-1 complex is transported to the Golgi apparatus, where SAH-2 is processed by the Dsc complex and rhomboid protease Rbd-2 to release the activated N-SAH-2, which enters the nucleus and activates genes required for ergosterol biosynthesis. Meanwhile, activated SAH-2 blocks the expression of genes (at some level) encoding extracellular oxygen-consuming enzymes (CDH-1 and LPMOs) and genes associated with an IRE-1/HAC-1-independent UPR pathway to limit the consumption of oxygen in surrounding cells. The green arrows indicate the UPR pathway, the purple arrows indicate cellulase enzyme trafficking and secretion, and the light blue arrows indicate the SAH-2 (SREBP) pathway.

Chromatin immunoprecipitation sequencing (ChIP-seq) analysis of SrbA in *A. fumigatus* (30), combined with the expression pattern of ergosterol biosynthesis genes for *N. crassa* (Fig. 3A), supports the hypothesis that SAH-2 directly regulates *erg-1*, *erg-3*, *erg-5*, *erg-11*, and *erg-25* and that the increased expression of these direct target genes is a good indicator of SAH-2 activation. The expression of these genes was significantly higher in the M*clr-2* strain than in the WT, suggesting that SREBP was highly activated when cellulase trafficking and secretion were induced. However, the hypersecretion phenotype in the M^*clr-*2^ Δ*sah-2* strain was eliminated when the oxygen supply was increased in bioreactors (see Fig. S5C), suggesting that the production of cellulases in *N. crassa* might lead to a reduction of available oxygen. Importantly, comparative analyses of transcriptional profiles revealed that gene expression levels of oxygen-dependent extracellular enzymes like LPMOs, which are involved in cellulose deconstruction, were increased in the M^*clr-*2^ Δ*sah-2* strain. These data indicate that activation of SAH-2 has a negative impact on the expression of extracellular-oxygen-consuming enzymes, particularly cellulases that utilize molecular oxygen, suggesting feedback regulation of genes encoding oxygen-requiring enzymes that are not essential (Fig. 7).

In addition to genes encoding LPMOs, a set of genes associated with ER stress responses had higher expression levels in the M^*clr-*2^ Δ*sah-2* strain than in the M*clr-2* strain (see Table S2). This observation led us to test the interconnections among the UPR, the SREBP pathway, and cellulase secretion. Previously, it was reported that the UPR is triggered when fungal cells are exposed to recalcitrant carbon sources; that spliced *hac1* transcripts increase in *T. reesei* (31), *A. nidulans* (32), and *N. crassa* (26, 27) when they are grown on lignocellulose; and that a Δ*hac-1* mutant is deficient in the secretion of cellulases (26, 28). However, our RNA-seq data analyses showed that the increased expression of UPR-associated genes observed in the M^*clr-*2^ Δ*sah-2* mutant was apparently not the result of the classic IRE-1/HAC-1-mediated UPR pathway, since spliced *hac-1* levels were lower in the M^*clr-*2^ Δ*sah-2* strain (Fig. 6A). Additionally, of the 176 protein secretion-associated genes that showed increased expression levels in the M^*clr-*2^ Δ*sah-2* strain, only 6 (NCU08707, NCU10051, NCU09485, NCU09265, NCU03982, and NCU08607) were identified within the HAC-1 regulon in *N. crassa* (26). Our data raise the possibility that a *hac-1*-independent UPR pathway exists in *N. crassa* and that this pathway is repressed (at some level) by SAH-2 (Fig. 7). The hypothesis was supported by the observation that a Δ*hac-1* Δ*sah-2* mutant showed normal secretion of cellulases and utilization of cellulose. The UPR in mammals uses at least two additional ER-resident sensors that are thought to act in parallel to induce downstream UPR targets. These include ATF-6 and the ER transmembrane kinase PERK (33, 34). Even though homologs of ATF-6 or PERK are not present in the genomes of yeast or filamentous fungi, our data suggest the presence of a second regulatory mechanism for the fungal UPR pathway that involves SAH-2. In mammalian cells, ER stress induces SREBP activation, although molecular mechanisms associated with cross talk between these two pathways may occur by several mechanisms that are not well elucidated (35). Additionally, it raises the question of the relationship of the UPR and the SREBP pathway for functions in filamentous fungi not associated with sterol homeostasis, such as virulence. Future characterization of the regulatory network of the UPR and SREBP pathways will be very informative for deciphering cellulase trafficking and lignocellulose deconstruction in filamentous fungi. This knowledge can benefit rational strain engineering to improve the production of lignocellulose-degrading enzymes and other biomass-based bioproducts.

## MATERIALS AND METHODS

### Strains and strain construction.

*N. crassa* WT strain OR74A (FGSC2489) and available single-gene deletion mutants were obtained from the Fungal Genetics Stock Center (http://www.fgsc.net/). The Δ*scp-1* mutant was constructed by transforming WT strain OR74A with a 3.8-kbp DNA fragment containing a 1.3-kbp flanking region homologous to the sequence upstream of *scp-1*, a selectable marker (*trpC* promoter-driven hygromycin B phosphotransferase) (16), and a 1.1-kbp flanking sequence homologous to the downstream region of *scp-1*. Transformants were selected for resistance to hygromycin and tested for genotypes by diagnostic PCR with primers Vscp1-F (GATAGTAGCTTCCGAGAACTTGTC) and hph-R (GGCCGCATAACAGCGGTCATTGAC). The Δ*sre-2* mutant was constructed by transforming WT strain OR74A with a 3.7-kbp DNA fragment containing a 1.1-kbp flanking region homologous to the upstream region of *sre-2*, the hygromycin B-selectable marker, and a 1.2-kbp flanking region homologous to the downstream region of *sre-2*. Transformants were selected for resistance to hygromycin and tested for genotypes by diagnostic PCR with primers VsreB-F (CTTGACCTGCTCGACGAAAACACAC) and hph-R (GGCCGCATAACAGCGGTCATTGAC). The M^*clr-*2^ Δ*erg-25* strain was constructed by transforming the M*clr-2* (*his-3*::P*ccg-1*-*clr-2 rid-1* Δ*sad-1*) strain (17) with a 3.4-kbp DNA fragment containing a 1.2-kbp flanking region homologous to the sequence upstream of *erg-25*, the *trpC* promoter-driven nourseothiricin acetyltransferase *nat-1* gene from *Streptomyces noursei* (36), and a 1.2-kbp flanking region homologous to the downstream region of *erg-25*. Transformants were selected for resistance to nourseothricin and tested for genotypes by diagnostic PCR with primers V*erg25*-F (GTGATGATCGATGACAGGATTGG) and nat-R (GGTGAAGGACCCATCCAGTG). Fifty micromolar SCR7 (37), which is an inhibitor of nonhomologous end joining, was used to increase the homologous recombination ratio of all transformations. Positive transformants were backcrossed to FGSC 4200 to obtain *Δscp-1* mutant, *Δsre-2* mutant, and M^*clr-*2^ Δ*erg-25* homokaryotic strains.

The M^*clr-*2^ Δ*hac-1* strain was created by transforming the M*clr-2* strain with a 3.4-kbp DNA fragment containing a 1.2-kbp flanking region homologous to the sequence upstream of *hac-1*, a *trpC* promoter-driven nourseothiricin acetyltransferase *nat-1* gene, and a 1.2-kbp flanking region homologous to the region downstream of *hac-1*. The M^*clr-*2^ Δ*hac-1* Δ*sah-2* strain was created by transforming the same fragment described above into the M^*clr-*2^ Δ*sah-2* strain. SCR7 (50 µM) (37) was used for these two transformations. Transformants were selected for resistance for nourseothricin and tested for genotypes by diagnostic PCR with primers V*hac-1*-F (TGGTAGCTTTGGGAGATATTGC) and nat-R (GGTGAAGGACCCATCCAGTG). Homokaryotic M^*clr-*2^ Δ*hac-1* and M^*clr-*2^ Δ*hac-1* Δ*sah-2* strains were obtained by microconidial purification as described elsewhere (http://www.fgsc.net/fgn37/ebbole1.html). The M^*clr-*2^ Δ*sah-*2, M^*clr-*2^ Δ*dsc-1*, M^*clr-*2^ Δ*scp-1*, M^*clr-*2^ Δ*erg-3*, and M^*clr-*2^ Δ*erg-24* strains were generated by crosses.

VMM (38) was used to grow *N. crassa*. Conidia were inoculated into 100 ml of liquid medium at 10^6^ conidia/ml and grown at 25°C on a rotary shaker (200 rpm) for the times indicated in constant light. Synthetic cross medium (39) was used for crosses. For hypoxic conditions, a hypoxia incubation chamber (Invivo2 400; Ruskinn) was used. The chamber was maintained at 25°C and filled with N_2_ with 0.5% O_2_.

### Protein and enzyme assays.

The protein concentration in supernatants was determined by the Bradford method (Bio-Rad Protein Assay). Endoglucanase activity was determined by with azo-carboxymethyl cellulose (Azo-CM-Cellulose; Megazyme) in accordance with the manufacturer’s specifications. CDH-1 activity was measured by monitoring the time-dependent reduction of 300 μM 2,6-dichloroindophenol (DCIP) at a wavelength of 520 nm in 100 mM sodium acetate, pH 5.0, containing 30 mM cellobiose (22). The reaction was started by adding 180 μl of the DCIP-based assay solution to a 20-μl supernatant sample in 96-well plates and monitored in a plate reader at 30°C for 5 min. One unit is equivalent to the conversion of 1 μmol min^−1^. CBH-1 activity was measured with soluble 4-methylumbelliferyl-β-d-cellobiose (Sigma) as the substrate as previously described (40).

### Sterol extraction and GC-MS analysis.

Fungal biomass from cultures was filtered and dried in a 65°C oven for 2 days. One hundred milligrams of dry biomass of each sample was ground into powder for extraction of sterol, and GC-MS was used to analyze the sterol components by suspension in a solution consisting of 4 ml of methanol and 2 ml of 60% (wt/vol) KOH supplemented with 10 μg of cholesterol as a recovery internal standard. Esters were saponified by incubation at 75°C with agitation for 4 h. Nonsaponifiable sterols were extracted twice with 4 ml of petroleum ether and evaporated with a stream of nitrogen gas to dry. The dry extract was resuspended in 1 ml of toluene. A 50-ml sample was added to 50 µl of *N*,*O*-bis(trimethylsilyl)trifluoroacetamide and incubated at 70°C for 30 min. The trimethylsilyl ether derivatization generated by this step was used for GC-MS analysis (41).

GC-MS analyses were performed with a CP-3800 gas chromatograph equipped with a FactorFour capillary column (VF-5 ms; 15-m length, 0.32-mm inside diameter, 0.25-μm film thickness) and analyzed with a Varian 320-MS triple-quadrupole mass spectrometer. Electron impact MS (70 eV, scanning from 80 to 600 at 1-s intervals) was performed with He as the carrier gas (30 cm/s), a detector temperature of 160°C, and column temperatures of 200 to 330°C (200°C for 1 min, 20°C/min to 330°C, and then held for 3 min). All injections were run in a splitless mode, and the injection volume was 1 μl. Sterol species were identified by comparison with standards or with MS data in the library. Peak areas for each sterol were normalized to the internal standard cholesterol peak area to correct for recovery.

### RNA-seq and data analysis.

Mycelial biomass from 24-h VMM cultures previously transferred from a 16-h VMM culture was filtered and frozen in liquid nitrogen for RNA extraction. RNA-seq libraries were prepared in accordance with standard protocols from Illumina Inc. (San Diego, CA) and sequenced on the Illumina Genome Analyzer IIx and HiSeq 4000 platforms at the genome center of the University of California Davis. Raw RNA sequencing reads (fastq files) were first subjected to FASTX toolkit (http://hannonlab.cshl.edu/fastx_toolkit/) to remove adaptor contamination and check quality. Reads passing this filter were mapped against *N. crassa* predicted transcripts from the OR74A genome v 12 (*Neurospora crassa* Sequencing Project, Broad Institute of Harvard and MIT [http://www.broadinstitute.org/]) with Tophat v 2.0.4. Numbers of RPKM were calculated with Cufflinks v 2.2.1, and differential expression analyses were performed with DESeq2 package version 1.10.1. The cluster 3.0 software suite (http://bonsai.hgc.jp/~mdehoon/software/cluster/software.htm) was used to perform hierarchical clustering analysis. The Munich Information Center for Protein Sequences FunCat online tool (http://mips.helmholtz-muenchen.de/funcatDB/) was used for functional classification of genes of interest.

### Bioreactor fermentation.

Conidia at 10^6^/ml were inoculated into a parallel 1-liter stirred tank bioreactor (DASGIP Bioreactor system, type DGCS4; Eppendorf AG, Germany) containing 500 ml of VMM and incubated for 48 h. The pH was set at 5.5 by the addition of 1 M HCl or 1 M KOH solution, and the temperature was maintained at 25°C. The DO tension was initially calibrated at 100%, and the DO level was controlled by manipulating the airflow or the stirrer speed. For a lower DO supply, the aeration flow rate was set at 30 liters/h and the stirrer speed was set as 400 rpm. For a higher DO supply, an automatic DO control system was used, with an aeration flow rate of up to 50 liters/h and a stirrer speed of up to 800 rpm when the DO level was lower than 40% of that at the beginning. During fermentation, 3-ml samples were taken every 12 h for measurement of the secreted protein concentration and enzyme activity.

### Statistical significance tests.

Statistical significance was determined by *t*-test analysis by the false discovery rate (FDR) approach. Asterisks indicate significant differences (*, *P* < 0.05; **, *P* < 0.01; ***, *P* < 0.001). ns, not significant.

### Accession number(s).

The data discussed in this publication have been deposited in NCBI’s Gene Expression Omnibus (42) and are accessible through GEO Series GenBank accession no GSE95807.
