# Supplementary figures and images for: Deciphering the Regulatory Network between the SREBP Pathway and Protein Secretion in Neurospora crassa
Source: mBio. 2017 Apr 18;8(2):e00233-17. doi: 10.1128/mBio.00233-17 (PMC5395666; doi:10.1128/mBio.00233-17)

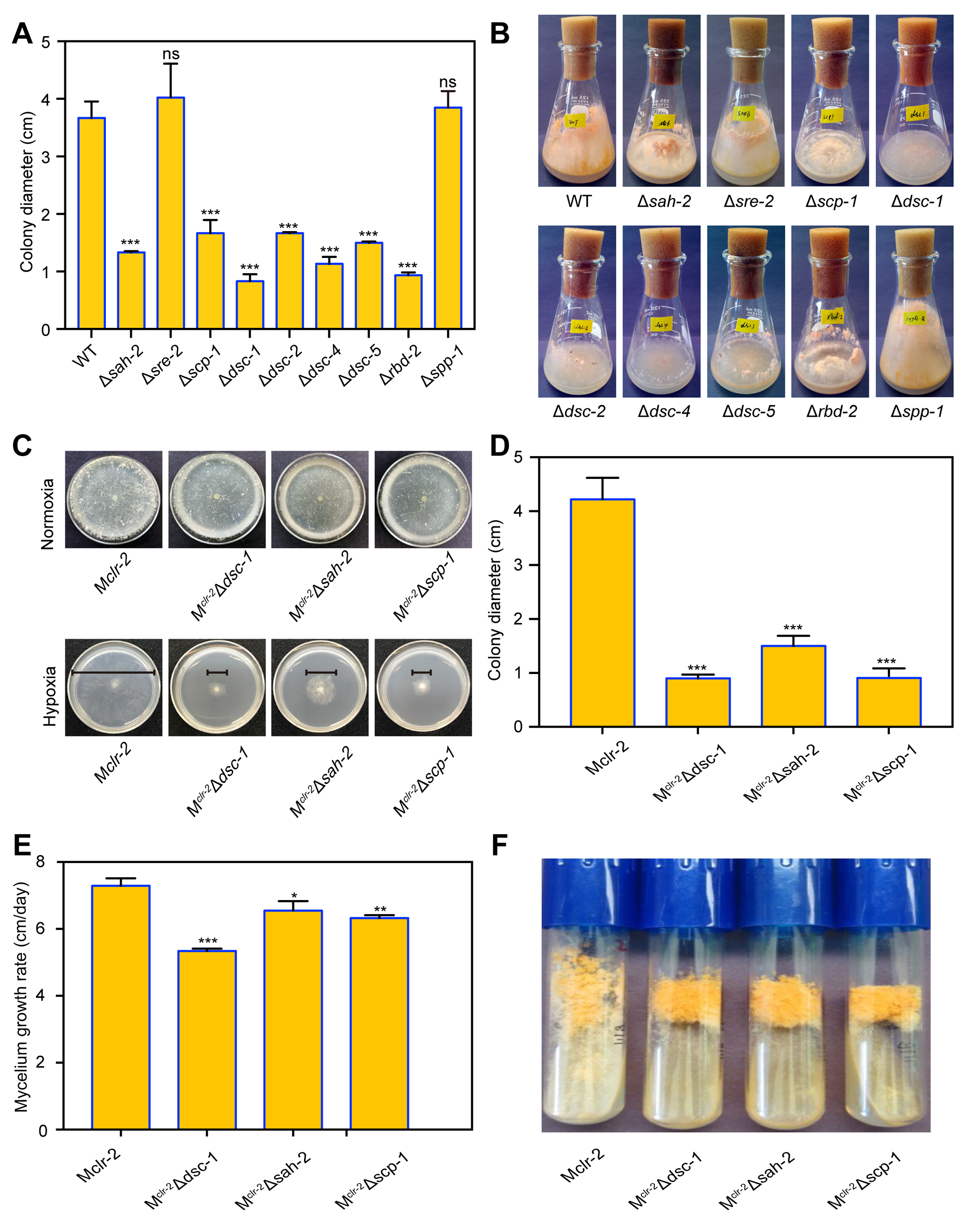

Supplement: FIG S1 [file mbo002173281sf1.tif]

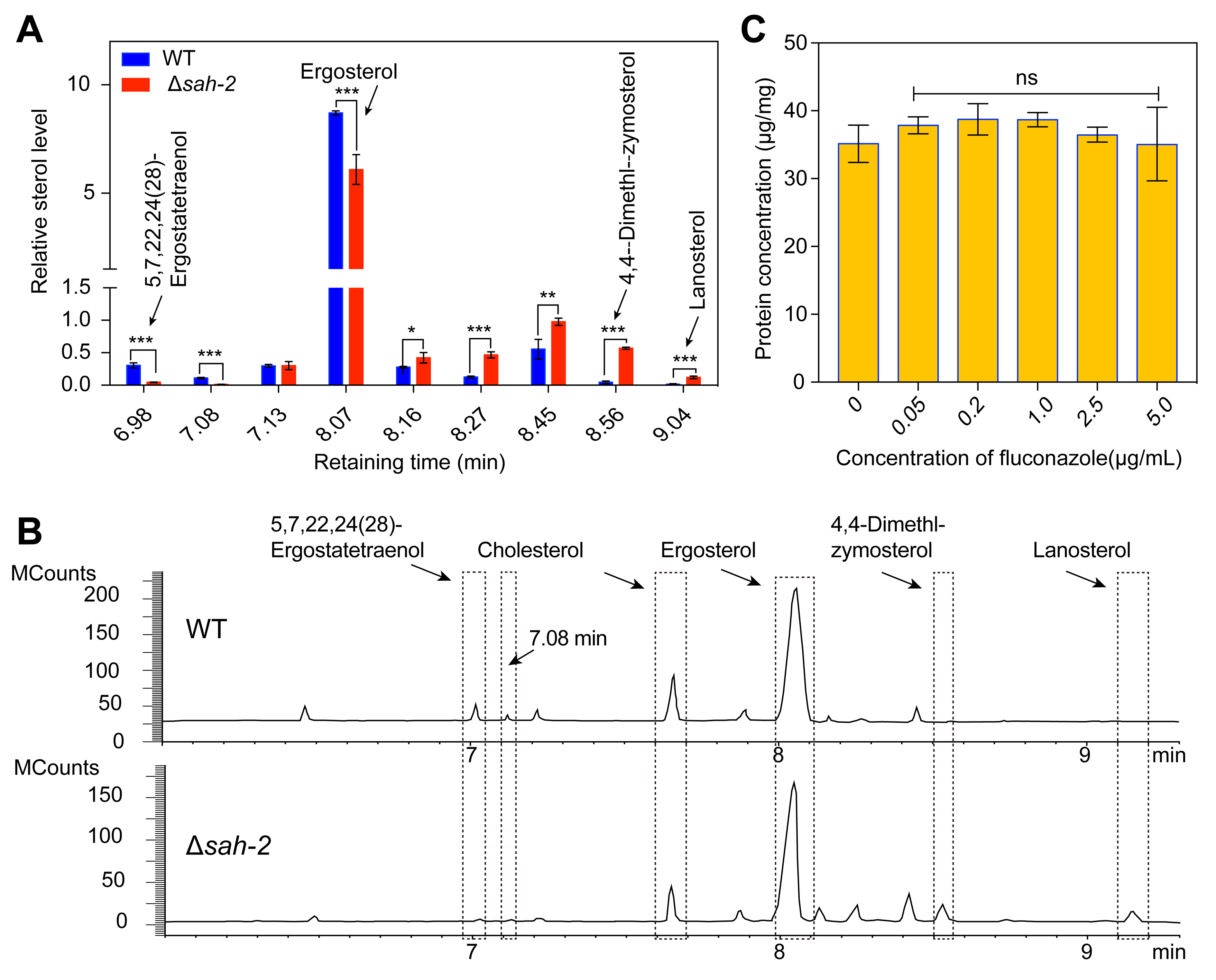

Supplement: FIG S2 [file mbo002173281sf2.tif]

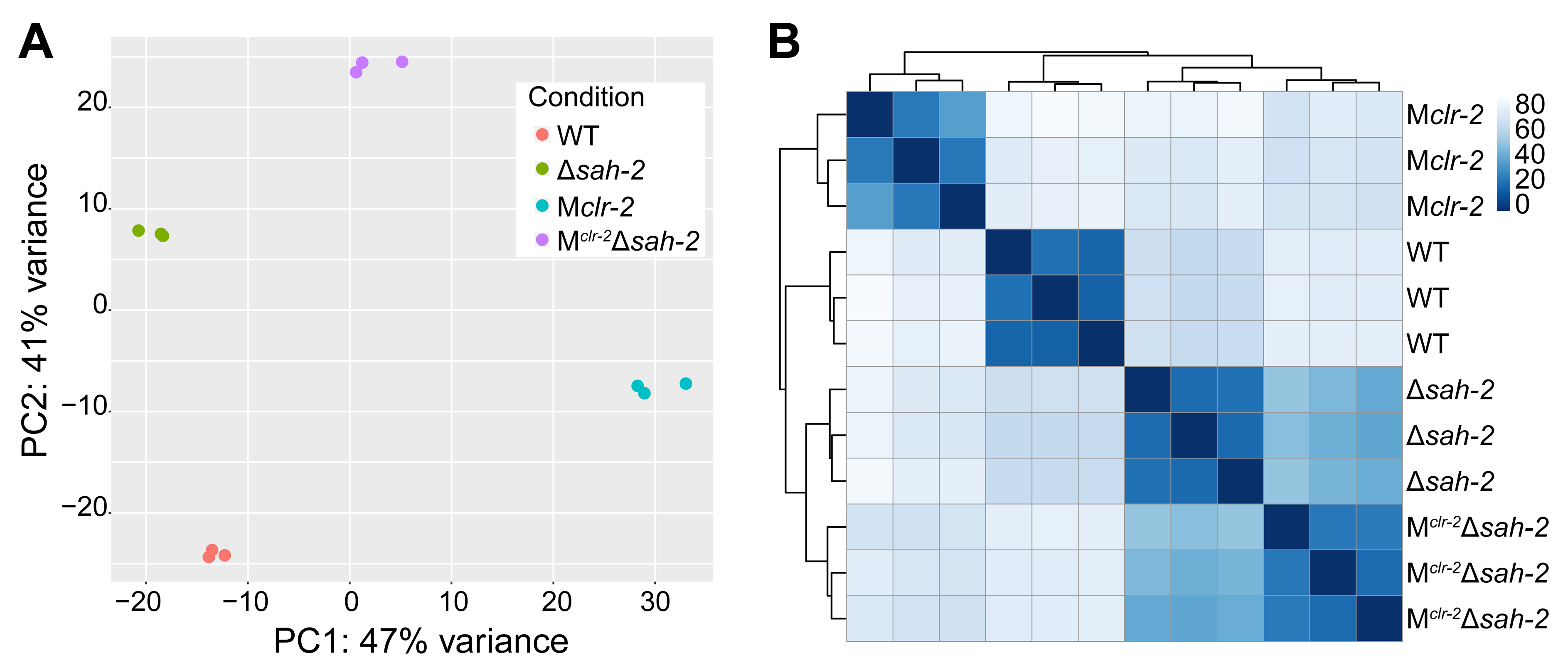

Supplement: FIG S3 [file mbo002173281sf3.tif]

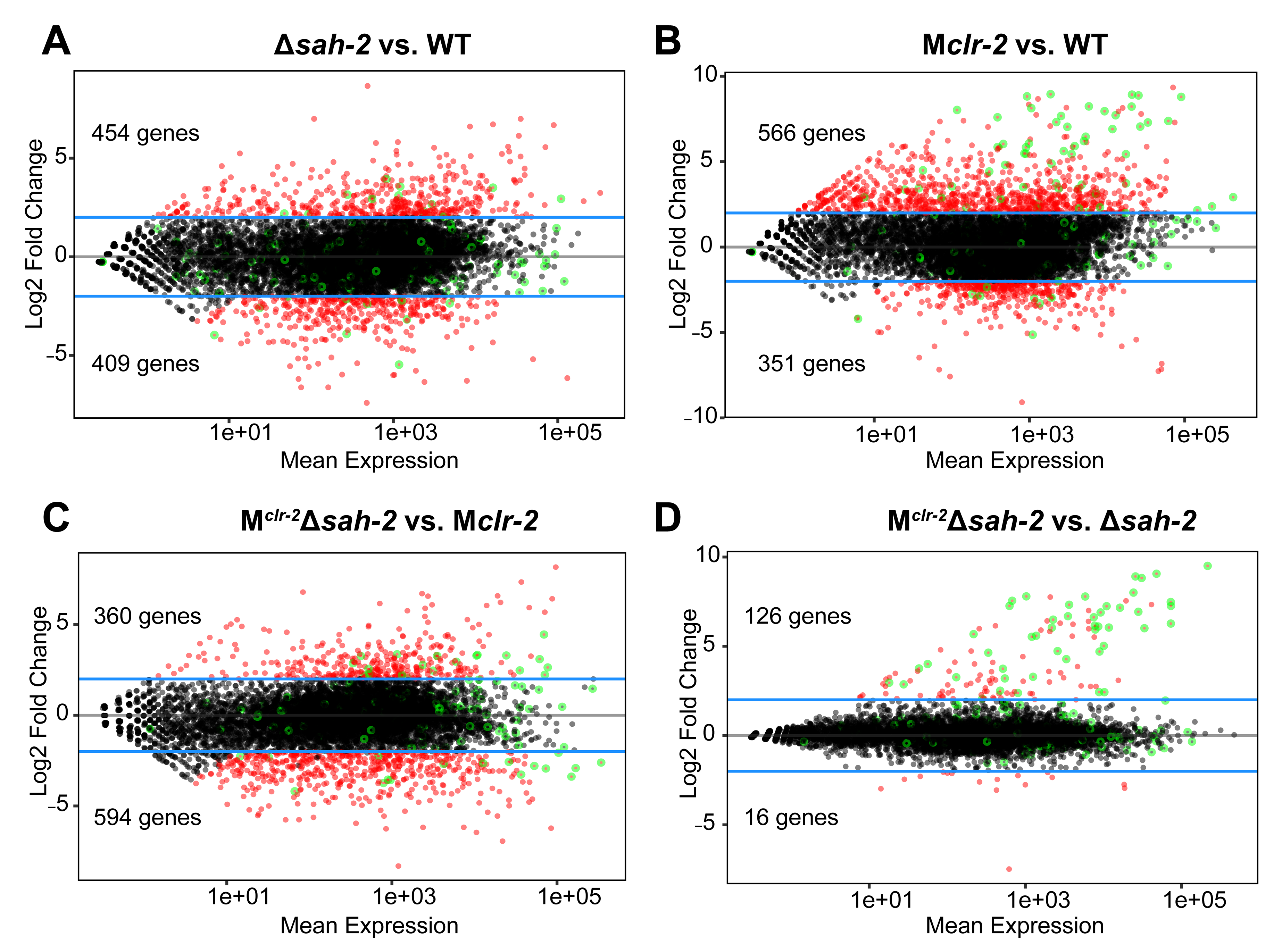

Supplement: FIG S4 [file mbo002173281sf4.tif]

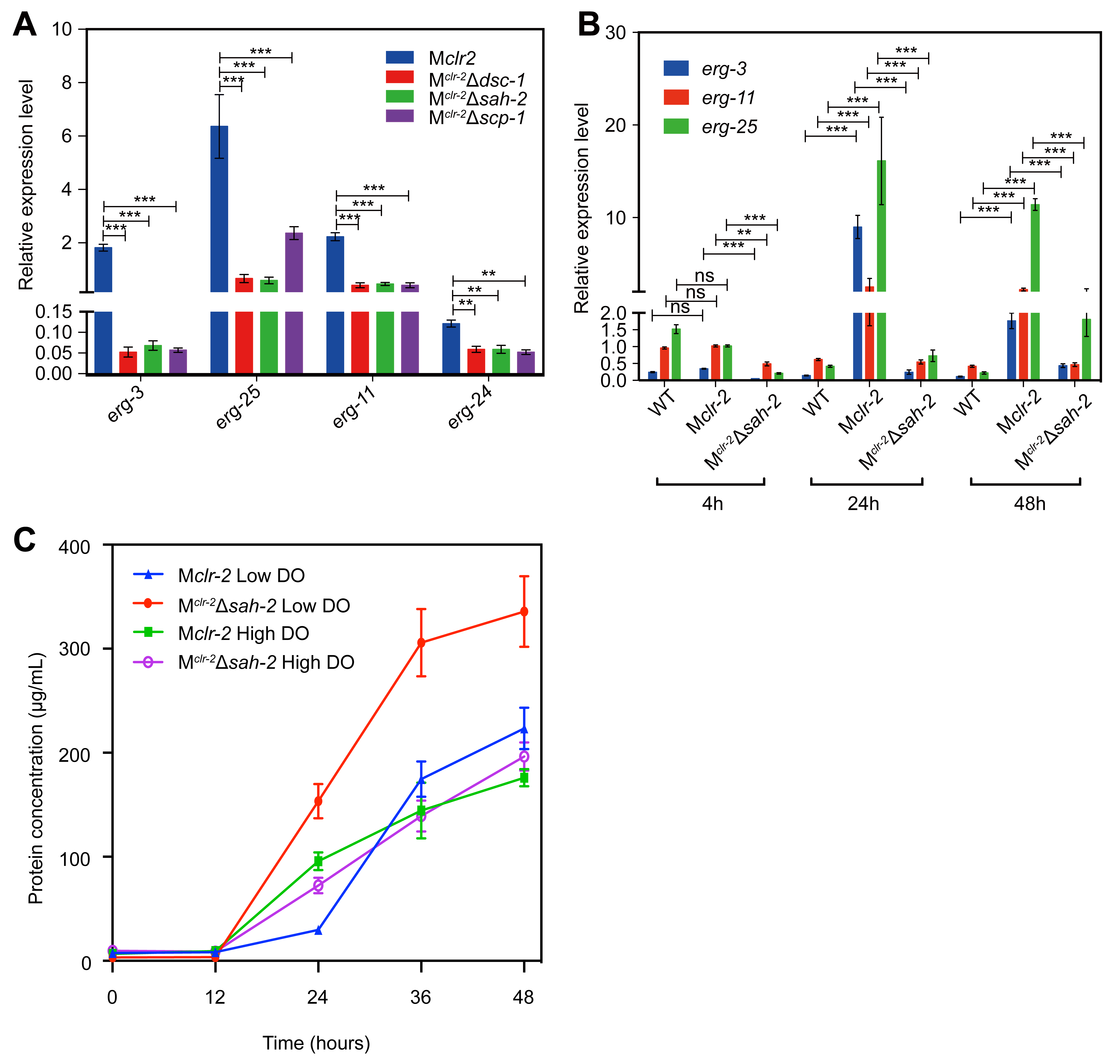

Supplement: FIG S5 [file mbo002173281sf5.tif]
