# Supplementary material for: Deciphering the Regulatory Network between the SREBP Pathway and Protein Secretion in Neurospora crassa
Source: mBio. 2017 Apr 18;8(2):e00233-17. doi: 10.1128/mBio.00233-17 (PMC5395666; doi:10.1128/mBio.00233-17)
Supplement: TABLE S1 [file mbo002173281st1.docx]

| **Locus** | **Function** | **RPKM** | | | | **Fold Change** |
| --- | --- | --- | --- | --- | --- | --- |
|  |  | **WT** | **M*clr-2*** | **Δ*sah-2*** | **M*^clr-2^Δsah*-2** | **M*^clr-2^Δsah*-*2*/M*clr-2*** |
| NCU00206 | Cellobiose dehydrogenase CDH-1 | 2.46 | 70.91 | 2.36 | 853.85 | 12.04 |
| NCU07760 | Lytic polysaccharide monooxygenase | 1.25 | 16.16 | 1.48 | 93.17 | 5.76 |
| NCU05121 | Endoglucanase V | 3.79 | 100.22 | 2.92 | 946.83 | 9.45 |
| NCU07190 | Exoglucanase 3 | 5.52 | 153.11 | 6.62 | 4468.13 | 29.18 |
| NCU07520 | Lytic polysaccharide monooxygenase | 0.31 | 2.06 | 0.25 | 37.21 | 18.02 |
| NCU00836 | Lytic polysaccharide monooxygenase | 0.98 | 33.29 | 0.32 | 161.82 | 4.86 |
| NCU02240 | Lytic polysaccharide monooxygenase | 8.32 | 606.91 | 3.36 | 2150.60 | 3.54 |
| NCU07898 | Lytic polysaccharide monooxygenase | 2.70 | 389.79 | 1.62 | 2194.85 | 5.63 |
| NCU05969 | Lytic polysaccharide monooxygenase | 6.34 | 15.44 | 7.25 | 22.15 | 1.43 |
| NCU09680 | Cellobiohydrolase CBHII | 9.85 | 422.00 | 10.01 | 1844.69 | 4.37 |
| NCU05924 | Endo-1,4-beta-xylanase | 2.75 | 595.44 | 3.16 | 4799.77 | 8.06 |
| NCU02855 | Endo-1,4-beta-xylanase A | 4.67 | 661.97 | 4.30 | 5153.68 | 7.79 |
| NCU02916 | Lytic polysaccharide monooxygenase | 6.40 | 111.47 | 7.48 | 209.00 | 1.87 |
| NCU03328 | Lytic polysaccharide monooxygenase | 6.37 | 349.20 | 9.26 | 748.24 | 2.14 |
| NCU08760 | Lytic polysaccharide monooxygenase | 9.06 | 1622.38 | 8.02 | 5217.94 | 3.22 |
| NCU07326 | Hypothetical protein | 12.49 | 3659.46 | 12.27 | 11866.36 | 3.24 |
| NCU00972 | endo-1,4-beta-galactosidase | 4.09 | 223.29 | 3.82 | 640.49 | 2.87 |
| NCU08398 | Aldose 1-epimerase | 3.49 | 553.07 | 2.84 | 2091.22 | 3.78 |
| NCU09491 | Feruloyl esterase B | 6.03 | 1100.80 | 20.01 | 3790.23 | 3.44 |
| NCU09764 | Lytic polysaccharide monooxygenase | 1.15 | 7.73 | 3.84 | 54.37 | 7.03 |

**Table S1:** Gene expression levels of extracellular protein encoding genes with a higher

expression level in the M*^clr-2^*Δ*sah-2* strain relative to *Mclr-2* strain.
