# Supplementary material for: Deciphering the Regulatory Network between the SREBP Pathway and Protein Secretion in Neurospora crassa
Source: mBio. 2017 Apr 18;8(2):e00233-17. doi: 10.1128/mBio.00233-17 (PMC5395666; doi:10.1128/mBio.00233-17)
Supplement: TABLE S2 [file mbo002173281st2.docx]

| **Gene ID** | **Function** | **RPKM** | | | | | | | | | **Fold Change** |
| --- | --- | --- | --- | --- | --- | --- | --- | --- | --- | --- | --- |
|  |  | **WT** | | **M*clr-2*** | | **Δ*sah-2*** | | **M*^clr-2^Δsah*-*2*** | | | **M*^clr-2^Δsah*-*2* /M*clr-2*** |
| **Protein Folding** | | | | | | | | | | | |
| NCU01856 | transcriptional activator HAC-1 | 258.73 | | 122.24 | | 88.53 | | 130.57 | | | 1.07 |
| NCU02202 | serine/threonine kinase IRE-I | 57.77 | | 51.99 | | 24.34 | | 25.96 | | | 0.50 |
| NCU09485 | HSP70-6 chaperone dnaK | 28.03 | | 13.44 | | 20.76 | | 40.86 | | | 3.04 |
| NCU09223 | Protein disulfide isomerase | 138.44 | | 154.12 | | 119.09 | | 321.62 | | | 2.09 |
| NCU09265 | calreticulin | 136.07 | | 139.32 | | 120.61 | | 248.76 | | | 1.79 |
| NCU03982 | glucose regulated protein 78, GRP-78 | 171.41 | | 154.33 | | 152.20 | | 454.11 | | | 2.94 |
| NCU07188 | GTP cyclohydrolase-2 | 27.82 | | 10.62 | | 45.92 | | 56.41 | | | 5.31 |
| NCU00104 | heat shock protein 98, HSP-98 | 35.63 | | 58.16 | | 149.20 | | 191.33 | | | 3.29 |
| NCU04334 | chaperonin | 66.86 | | 81.48 | | 161.10 | | 158.64 | | | 1.95 |
| NCU04142 | heat shock protein 80, HSP-80 | 179.46 | | 324.84 | | 495.75 | | 534.82 | | | 1.65 |
| NCU01589 | heat shock protein 60, HSP-60 | 47.43 | | 28.46 | | 141.53 | | 133.50 | | | 4.69 |
| NCU03853 | peptidyl-prolyl cis-trans isomerase | 40.46 | | 17.83 | | 53.48 | | 38.33 | | | 2.15 |
| NCU05269 | heat shock protein 88, HSP88 | 48.98 | | 23.37 | | 69.55 | | 49.05 | | | 2.10 |
| **Protein translocation** | | | | | | | | | | | |
| NCU00169 | translocation component SEC-63 | | 54.89 | | 53.83 | | 42.63 | | 92.64 | 1.72 | |
| NCU02681 | translocation protein EC-71 | | 56.99 | | 42.33 | | 53.49 | | 103.41 | 2.44 | |
| NCU07746 | translocation complex subunit SEC-72 | | 71.35 | | 41.57 | | 43.29 | | 94.70 | 2.28 | |
| NCU06677 | microsomal signal peptidase subunit | | 72.81 | | 58.28 | | 64.34 | | 101.98 | 1.75 | |
| NCU01146 | signal sequence receptor alpha chain | | 90.11 | | 131.69 | | 159.57 | | 211.61 | 1.61 | |
| **Protein glycosylation** | | | | | | | | | | | |
| NCU03503 | mannosyltransferase, COT-5 | | 30.97 | | 46.70 | | 49.58 | | 81.12 | 1.74 | |
| NCU06386 | dolichyl-phosphate beta-glucosyltransferase | | 18.30 | | 16.86 | | 20.58 | | 28.41 | 1.68 | |
| NCU10762 | UDP-N-acetyl-glucosamine-1-P transferase Alg7 | | 20.35 | | 21.92 | | 35.66 | | 36.22 | 1.65 | |
| NCU00669 | oligosaccharyl transferase subunit | | 55.26 | | 37.23 | | 40.87 | | 54.71 | 1.47 | |
| NCU10721 | UDP-galactose transporter | | 42.02 | | 20.09 | | 20.82 | | 42.91 | 2.14 | |
| NCU02778 | 1,2-alpha-mannosidase | | 20.98 | | 10.15 | | 16.46 | | 17.30 | 1.70 | |
| NCU07067 | mannosyl-oligosaccharide alpha-1,2-mannosidase | | 48.78 | | 61.91 | | 92.22 | | 295.48 | 4.77 | |
| **Vesicle trafficking/transport** | | | | | | | | | | | |
| NCU04293 | COPII-coated vesicle protein | | 30.65 | | 19.77 | | 21.21 | | 35.43 | 1.79 | |
| NCU08607 | endoplasmic reticulum-Golgi intermediate compartment protein 3 | | 60.14 | | 56.87 | | 58.30 | | 97.98 | 1.72 | |
| NCU03387 | vesicular-fusion protein SEC-18 | | 26.45 | | 6.34 | | 29.58 | | 23.08 | 3.64 | |
| NCU01644 | intracellular protein transporter USO-A | | 17.79 | | 6.92 | | 13.09 | | 13.43 | 1.94 | |
| **Proteolytic degradation** | | | | | | | | | | | |
| NCU03028 | deubiquitination-protection protein DPH-1 | | 54.04 | | 23.31 | | 42.18 | | 40.41 | 1.73 | |
| NCU08445 | misfolded glycoproteins degradation protein YOS-9 | | 35.97 | | 20.75 | | 18.82 | | 32.81 | 1.58 | |
| NCU06603 | ThiJ/PfpI family protein | | 192.53 | | 135.32 | | 425.19 | | 526.31 | 3.89 | |
| NCU01479 | matrix AAA protease MAP-1 | | 32.41 | | 25.64 | | 64.82 | | 69.68 | 2.72 | |
| NCU05261 | ATP-dependent protease La | | 12.32 | | 6.73 | | 31.08 | | 21.31 | 3.17 | |
| NCU02113 | ubiquitin-conjugating enzyme E2 | | 78.94 | | 40.38 | | 102.39 | | 119.79 | 2.97 | |
| NCU10477 | ubiquitin-conjugating enzyme E2 | | 83.83 | | 77.85 | | 122.07 | | 116.25 | 1.49 | |
| NCU10046 | ubiquitin conjugating enzyme UBC-8 | | 116.16 | | 54.96 | | 187.73 | | 178.45 | 3.25 | |

**Table S2:** Gene expression levels of genes involved in ER stress response in WT, M*clr-2*,

Δ*sah-2* and M*^clr-2^*Δ*sah-2* strains.
